# Adaptation and validation of the short version WHOQOL-HIV in Ethiopia

**DOI:** 10.1186/s13033-016-0062-x

**Published:** 2016-04-09

**Authors:** Markos Tesfaye, Mette Frahm Olsen, Girmay Medhin, Henrik Friis, Charlotte Hanlon, Lotte Holm

**Affiliations:** Department of Psychiatry, College of Health Sciences, Jimma University, Jimma, Ethiopia; Department of Nutrition, Exercise and Sports, Faculty of Science, University of Copenhagen, Copenhagen, Denmark; Aklilu Lemma Institute of Pathobiology, Addis Ababa University, Addis Ababa, Ethiopia; Department of Psychiatry, School of Medicine, College of Health Sciences, Addis Ababa University, Addis Ababa, Ethiopia; Centre for Global Mental Health, Institute of Psychiatry, King’s College London, London, UK; Department of Food and Resource Economics, Faculty of Science, University of Copenhagen, Copenhagen, Denmark

**Keywords:** Quality of life, HIV, Cross-cultural validation, Ethiopia, WHOQOL-HIV bref

## Abstract

**Background:**

Quality of life of patients is an important element in the evaluation of outcome of health care, social services and clinical trials. The WHOQOL instruments were originally developed for measurement of quality of life across cultures. However, there were concerns raised about the cross-cultural equivalence of the WHOQOL-HIV when used among people with HIV in Ethiopia. Therefore, this study aimed at adapting the WHOQOL-HIV bref for the Ethiopian setting.

**Methods:**

A step-wise adaptation of the WHOQOL-HIV bref for use in Ethiopia was conducted to produce an Ethiopian version—WHOQOL-HIV-BREF-Eth. Semantic and item equivalence was tested on 20 people with HIV. One hundred people with HIV were interviewed to test for measurement equivalence (known group validity and internal consistency) of the WHOQOL-HIV-BREF-Eth. Confirmatory factor analysis was conducted using data from 348 people with HIV who were recruited from HIV clinics.

**Results:**

In the process of adaptation, new items of relevance to the context were added while seven items were deleted because of problems with acceptability and poor psychometric properties. The Cronbach’s α for the final tool with twenty-seven items WHOQOL-HIV-BREF-Eth was 0.93. All six domains discriminated well between symptomatic and asymptomatic people with HIV (p < 0.001). Using confirmatory factor analysis, a second order factor structure with six first order indicator factors demonstrated moderate fit to the data ((χ^2^ = 627.75; DF = 259; p < 0.001), CFI = 0.82, TLI = 0.77 and RMSEA = 0.064).

**Conclusion:**

The WHOQOL-HIV-BREF-Eth has been shown to be a valid measure of quality of life for use in clinical settings among people with HIV in Ethiopia.

## Background

“Quality of life” is defined by the World Health Organization (WHO) as “a person’s perception of his/her position in life within the context of the culture and value systems in which he/she lives and in relation to his/her goals, expectations, standards and concerns” [1]. Measurement of quality of life (QoL) has gained more focus in recent years [2]. Quantifying differences in QoL between different groups of people and how they change over the course of time is of interest in its own right [3]. Furthermore, measures of QoL have been in special focus in the evaluation of the outcome of health care and social services [4]. They also provide the opportunity to obtain evidence of the impact of interventions from the patients’ perspective [5]. Therefore, QoL is considered to be an important aspect of the outcomes to be measured in effectiveness research [6].

Various tools have been developed and used for measurement of QoL [5]. One of the most frequently used measures of QoL is the WHOQOL-100, developed by the WHO two decades ago [1]. An HIV disease specific module for measuring QoL among people with HIV, WHOQOL-HIV, was developed as a cross-cultural instrument [7, 8]. The shorter version of WHOQOL-HIV, namely WHOQOL-HIV bref, was also validated through an international field trial [9]. Nevertheless, the WHO discourages simple translation of the WHOQOL instruments into a new language without prior cultural adaptation [1, 10]. Furthermore, researchers in the field have emphasized that cross-cultural adaptation of a QoL measurement tool is a prerequisite before undertaking any comparative studies of QoL due to varying conceptualisations of QoL in different sociocultural contexts [11].

The lack of a standardized approach to cross-cultural adaptation of QoL tools has been indicated previously [11], but various steps in cross-cultural adaptation of QoL measurement tools have been recommended [1, 11, 12]. In one model for cross-cultural adaptation of QoL tools, six elements of equivalence have been suggested [12]. *Semantic equivalence* refers to whether the items in the QoL tool have an equivalent meaning in the new language. This can be achieved through translation and back-translation by teams of translators and expert consensus meetings. *Conceptual equivalence* refers to whether the values a given population places on the different domains of QoL are similar in the setting. *Item equivalence* examines whether the items used to measure the QoL domains are relevant in the new setting to the concepts being measured. *Operational equivalence* addresses issues related to the mode of administration of the QoL measurement, e.g. face-to-face interviews or self-administered questionnaires. *Measurement equivalence* refers to the extent to which the psychometric properties of the QoL tool in the new setting are similar to the original tool. Measurement equivalence can be explored through assessment of internal consistency, known group validity and construct validity. Finally, *functional equivalence* looks at how successfully the QoL instrument measures what it is supposed to measure, namely the overall result of the various equivalences [12].

During the development of both the WHOQOL-100 and the WHOQOL-HIV, attempts were made to evaluate cross-cultural validity in selected international sites [1, 7, 8]. Subsequently, many researchers have validated the various versions of the WHOQOL HIV module in their own settings [13–22]. However, in Ethiopia, previous studies measuring QoL among various population groups have only conducted translation and back-translation of QoL tools without further adaptation [23–25]. Therefore, we conducted a more thorough adaption of WHOQOL-HIV bref in Ethiopia, aiming to address important aspects of cross-cultural equivalence.

Previously, our group conducted a qualitative study explore the conceptual equivalence of the WHOQOL-HIV tool among people with HIV in Jimma, Ethiopia [26]. Limitations of the conceptual basis for WHOQOL-HIV in the Ethiopian setting were identified. In particular, the WHOQOL-HIV did not appear to capture locally relevant themes such as disease disclosure, family responsibilities, exclusion from common resources, availability of adequate food and job opportunities [26].

Building on this work, we went on to conduct a step-wise cross-cultural adaptation of the WHOQOL-HIV bref based on recommendations from the WHOQOL Group and other experts in the field [1, 12]. In this paper, we describe the process of adaptation and validation of the Ethiopian version of WHOQOL-HIV bref designed for measuring QoL in clinical setting among people with HIV.

## Methods

### Site of the study

The study was conducted within Jimma University Specialized Hospital (JUSH), located in Jimma City, and two primary care centres (one in Jimma City, the other in Agaro town). The hospital and the primary care centers have HIV clinics providing services to people living with HIV who are residents of Jimma City and the surrounding Jimma sub-region in southwest Ethiopia.

### Study design

The design of the study took into consideration the recommendations of the WHOQOL Group [1] and followed the procedure of developing and validating a QoL instrument as proposed by Herdman and colleagues [12]. This involved investigation of *semantic*, *item,measurement and functional equivalences*.

### Study sample

#### Sample for item equivalence

A consecutive sample of 20 adults with HIV was recruited from the HIV clinic of JUSH in July 2009.

#### Sample for known-group validity

A further sample of 100 people with HIV (50 asymptomatic and 50 symptomatic/AIDS) was recruited from the consecutive attendees at the out-patient clinic at JUSH during October and November 2009.

#### Sample for confirmatory factor analysis (CFA)

Three hundred forty-eight adults with HIV, who were eligible to start antiretroviral treatment, were recruited from out-patient HIV clinics of JUSH and two primary care centers between July 2010 and July 2012. This formed the sample for the confirmatory factor analysis. A detailed description of this sample is available elsewhere [27].

### Procedures

#### Addition of new items

Six new items were added to the original WHOQOL-HIV bref which has 31 items and thus, the new draft instrument, with 37 items, was named WHOQOL-HIV-BREF-PILOT1. The items were formulated using the same methods as the WHOQOL-HIV group [1, 7]. These new items were generated from a qualitative study conducted in the same setting [26] as well as recommendation by experts in the field [28].

#### Semantic equivalence

The English version of the WHOQOL-HIV-BREF-PILOT1 instrument was translated by two independent bilingual medical doctors into Amharic, the official language of Ethiopia. Translators referred to the definitions of the questionnaire’s facets (items) described by the WHOQOL group to make sure that the original concept underlying each item was maintained in the translation [1, 7]. The Amharic version of the questionnaire was then translated back to English by two bilingual lecturers working at the Department of Amharic language, Jimma University.

Two trilingual (English, Amharic, Afaan Oromo) medical doctors translated the instrument into Afaan Oromo, the official language of the Oromia region. The Afaan Oromo version was translated back to English by lecturers at the Afaan Oromo language Department of Jimma University. A consensus meeting of the translators and the principal investigator was held to discuss difficult items and ensure semantic validity. Difficult items in the translation were noted for further investigation.

#### Item equivalence

The principal investigator administered the Amharic version of WHOQOL-HIV-BREF-PILOT1 instrument using cognitive interviewing methods [29]. Participants were asked if they had understood the question. The need for repetition was noted as a proxy indicator of item difficulty. Once participants responded, they were prompted to explain their answers and give examples. The interviews also included questions such as: “Were there any questions that you found irrelevant?” “Were there any questions that you found confusing or difficult to answer?” and “Do you suggest any particular local word to express the concept?”. While conducting the interviews three aspects were used to identify problematic items. The first indicator was when the respondent disclosed that the meaning of the item or specific word was not clear. The second was when the respondent gave a response but failed to elaborate on what he/she understood from the question. Finally, the third aspect was when respondent gave examples that indicated there was mis-conceptualization of what the question was intended to elicit.

The interviewer made a note of problematic items in the WHOQOL-HIV-BREF-PILOT1. After the first 10 interviews, problematic items were reworded or examples were added to clarify the concept before conducting the remaining 10 interviews. The validity of the translated items was assessed by comparing the respondents’ explanation of their responses with the descriptions of the facets operationalized in the original WHOQOL instruments [7]. Six items were deleted on the basis of being “difficult to understand” by more than half of the participants and not possible to improve comprehensibility by rewording, as well as items appearing to be irrelevant and culturally sensitive. The decision to remove the items was made by the investigators in consultation with the translators. The modified draft instrument, with 31 items, was named WHOQOL-HIV-BREF-PILOT2.

#### Measurement equivalence

##### Known group validity

The ability of items and domains to discriminate between symptomatic and asymptomatic/AIDS cases was assessed. The domains were investigated for the differences in mean scores between the two groups. In addition, the score on each of the items was tested for its ability to discriminate between symptomatic and asymptomatic/AIDS cases among people with HIV. In both cases a p value less than 0.05 was considered significant. Items with non-significant differences between the two groups were subsequently removed.

##### Internal consistency

The internal consistencies (Cronbach’s alpha) for the WHOQOL-HIV-BREF-PILOT2, as well as for each of the domains, were calculated. An a priori cut-off of corrected item-total correlation less than <0.3 was used to remove an item with poor reliability in a step-wise manner.

##### Construct validity

After having removed four additional items which performed poorly based on the above criteria, the final tool namely, WHOQOL-HIV-BREF-Eth was tested for construct validity. Thus, confirmatory factor analysis of the items on WHOQOL-HIV-BREF-Eth was conducted against the six factor structure of the original WHOQOL-HIV bref using the data from 348 people with HIV described above.

### Data collection

For all the three different samples, data on socio-demographic characteristics were collected. For the known group validation study, two nurses working at the HIV clinic were trained in the administration of the tool by the principal investigator. As part of the training, inter-rater reliability testing was carried out on 10 people with HIV. The QoL interviewers were masked to the HIV clinical stage documentation.

Data used for the confirmatory factor analysis was collected by research nurses and has been described elsewhere [27].

### Data analysis

Data were double entered and cleaned using Epidata (EpiData Association, Odense, Denmark) and analysed using STATA/IC version 11.2 (StataCorp LP, College Station, USA). The items were categorized into six domains as suggested by previous research on WHOQOL-HIV bref [9]. The mean scores of domains were multiplied by four as suggested by the WHOQOL group. The known-groups validity of domains was assessed by comparing the mean scores of asymptomatic and symptomatic people with HIV using multiple linear regressions adjusting for the effects of age, sex, and literacy status. In addition, each item was analyzed to check how well it differentiated between the two groups by comparing the median scores using the Wilcoxon rank sum test. Statistical tests were considered significant whenever the p-value was less than 0.05. The correlation among the various domains of QoL instrument was estimated using Spearman rank correlation. As a measure of internal consistency, Cronbach’s alpha and corrected item-total correlations were calculated for each of the domains as well as for the total scale.

Confirmatory factor analysis was fitted using AMOS software version 20 to evaluate the fit of the six factor structure proposed in previous field trial [9] using the data collected with WHOQOL-HIV-BREF-Eth. Four fit indices i.e. Chi squares (χ^2^), Tucker-Lewis Index (TLI), Comparative Fit Index (CFI), and Root Mean Square Error Approximation (RMSEA) were reported as a measure of the overall model fit. TLI and CFI are expected to be above 0.9 and RMSEA is expected to be below 0.06 for a relatively good fitting model [30]. As a measure of importance of the indicators of the second order factor, the standardized regression coefficients were computed for all six domains. Statistical significance of the loadings of each item on its hypothesized factor was also assessed.

### Ethical considerations

Ethical clearance was obtained from the Ethical Review Committee of Jimma University. Written informed consent was sought from study participants before the interviews.

## Results

### Socio-demographic characteristics

The item equivalence sample included eight men and 12 women. Their age ranged between 20 and 48 years, and five were illiterate.

The known-groups validity sample included a majority of females (62.0 %). The mean age of the participants was 32.5 (SD = 7.9) years with over half of them being in the age group 25–34 years. Nearly a quarter of the participants (24.0 %) were illiterate. Only 11.0 % of the respondents lived in a rural area (Table 1).

**Table 1:** Characteristics of people with HIV included in the known-groups validity study (n = 100)

Variable	Number	%
Sex		
Male	38	38.0
Female	62	62.0
Age in years		
Mean (SD^a^)	32.5 (7.9)	(Range = 18–55)
15–24	11	11.3
25–34	50	51.6
35–44	26	26.8
≥45	10	10.3
Literacy		
Illiterate	24	24.0
Literate	76	76.0
Place of residence		
Urban	89	89.0
Rural	11	11.0

^a^Standard deviation

The confirmatory factor analysis sample also included a majority of women (66.7 %) and their mean age was 32.9 (SD = 8.8) years. Of the sample, 101 (29.0 %) were illiterate.

### New items

Six new items were included before translation. Three of these new items addressed the issue of food and nutrition. The other three related to the social life of people with HIV (Table 2).

**Table 2:** List of new items added to WHOQOL-HIV bref

Items asking about the extent of having certain problems	Not at all	A little	Moderately	Very much	Extremely
*In the past 2* *weeks, to what extent are you concerned that people might find out about your HIV status?*	5	4	3	2	1
*In the past 2* *weeks, how much did you worry about food in your daily life?*	5	4	3	2	1
*In the past 2* *weeks, how much did you worry that your medications will not work due to not eating adequate food?*	5	4	3	2	1
*In the past 2* *weeks, how much did you worry about not being able to support your family?*	5	4	3	2	1

Items asking about level of satisfaction with certain aspects of life	Very dissatisfied	Dissatisfied	Neither satisfied nor dissatisfied	Satisfied	Very satisfied
*In the past 2* *weeks, how satisfied are you with your relations to other people in your community?*	1	2	3	4	5
*In the past 2* *weeks, how satisfied are you with your access to adequate food and nutrition?*	1	2	3	4	5

### Semantic equivalence

Translation and back translation revealed some discrepancies between the original English version and the back-translated version. For six of the original items semantic equivalence was problematic, however, they were retained for evaluation of item equivalence. The details of the problematic items are described using the numbering system of the WHOQOL-HIV bref.

G.1 “…*how would you rate your quality of life*”

The local language version for “*quality of life*”, “*ye’nurowon dereja*”, appeared to convey “*financial status*” rather than general quality of life. The consensus meeting modified the phrase to “*ye’nurowon terat*” and a phrase referring to ‘*financial, social and health aspects’* was added.

F1.4 *“…to what extent do you feel that physical pain prevents you from doing what you need to do?*”

The local language version used a term “*ye’akal hemem*”, which did not differentiate between “*physical pain*” from “*physical illness*”. However, the consensus meeting could not identify a better term that is commonly used. Therefore it was left as it was.

F24.2 “…*to what extent do you feel your life to be meaningful?*”

The local language version equivalent for “*your life*” was “*nurowo*”. However, when this expression was back-translated, it was translated to “*living standard*”. Therefore, after some discussion, the local language version was modified to “*menor*”, meaning “*life or existence*”.

F5.3 *“…how well are you able to concentrate?*”

The translated local term for “concentrate” was “*tekuret mestet*” which literally means “*paying attention*”. The consensus meeting replaced this expression with a local term “*hasabwon mesebseb*”, meaning “*gathering your thoughts*”.

F16.1 “…*how safe do you feel in your daily life?*”

The translation of this question was difficult because the term “*safe*” did not have a commonly used equivalent in the local language. The suggested terms in the local language, “*dehninet/wastena*”, meaning “*wellness/security*”, came closest in meaning.

F21.1 *“…to what extent do you have the opportunity for leisure activities?*”

The translation of “*leisure activities*” was problematic because its meaning became “*exercise in your free time*”. During the consensus meeting, a better phrase “*meznagna ye’magegnet edel*” was used; this phrase means “*opportunity to relax and enjoy*”.

For all items that inquired “*how satisfied …?*” the respondents were, the local term equivalent to “*how satisfied …?*”, “*…min yahel rektewal?*”, was changed to “*…min yahel tedestewal?*”, meaning “*how happy were you about…?*”

### Item equivalence

The participants understood the new items without difficulty. The items from the original WHOQOL-HIV bref that were found to be problematic are described below.

G.1 “…*how would you rate your quality of life*”

This item was found to be difficult as six participants repeatedly asked for the question to be re-read to them. In addition, four participants said they did not understand the question at all. Some of the participants interpreted the question as “*how clean is your environment?*” After 10 interviews, the term “*quality of life*” was replaced by “*goodness of living*” for the remaining 10 interviews. The participants understood the modified item without difficulty.

F24.2 “…*to what extent do you feel your life to be meaningful?*”

Four participants needed the question to be read out again. However, they all understood it well when the word “*hiwot*” meaning “life” was replaced by the word “*menor*” meaning “living”. It was found out that people did not commonly use the former term.

F5.3 *“…how well are you able to concentrate?”*

Of the first 10 participants, six did not understand the question. One respondent explained their “*I had no such problem*” response as “…*because I have only one partner and I have not been thinking about other persons*”. Therefore, for the remaining 10 interviews an explanatory example, “*such as being able to focus while having conversations*”, was added. However, the item remained difficult in most cases. Consequently, the item had to be removed.

F16.1 “…*how safe do you feel in your daily life?*”

Six of the first 10 participants reported that they did not understand the question. An explanatory clause was added, “*such as being able to go away from home and get back home in peace*”. This helped the remaining 10 participants understand the item better. Nonetheless, four explained: “*It is up to the Almighty God*”.

F22.1 “*…how healthy is your physical environment?*”

Several respondents interpreted the question as referring to “*the number of healthy individuals in your community*”. The item was modified as “…*how good is your environment for health?”* The remaining participants had no difficulty in understanding the modified item.

F7.1 “*…are you able to accept your bodily appearance?*”

Three of the first 10 participants needed the question to be read again, and the remaining seven participants said they did not understand the item despite repetition by the interviewer. They needed some elaboration. Three of them replied that they did not check in the mirror. For the remaining 10 interviews the item was modified to “*…your body or physical appearance?*” However, the item remained difficult to most participants and therefore, the item was removed.

F18.1 “*…have you enough money to meet your needs?*”

This item was not problematic during the interviews. However, all of the 20 participants responded “*not at all*”. Consequently, this item was removed.

F20.1 “…*how available to you is the information that you need in your day*-*to*-*day life?*”

Of the first 10 participants, seven did not appear to understand the item as intended. “*Information*” was understood as something very high level or as something not relevant to them. It was not possible to come up with simple rewording. Additional explanation, “*how to find something or some place; like a farmer may need to know if there is going to be adequate rain* etc.” was used. Although the latter version improved the clarity of the item, nine of the remaining 10 participants expressed that it was not relevant to them. Therefore, the item was removed.

F21.1 “*…to what extent do you have the opportunity for leisure activities?*”

Four participants did not understand the item in the sense it was intended. Five respondents understood the item to have a negative connotation such as “*hanging around*” and “*drinking alcohol*”. The item was modified to include the example “*such as chatting with people you know/friends*”. The modified item became clearer for the remaining 10 interviews.

F15.3 “*…how satisfied are you with your sex life?*”

This is was a culturally very sensitive item particularly for female participants. Many were embarrassed to be asked the question. In addition, most participants replied that they had stopped being sexually active after they became aware of their HIV status. The item was deleted due to it being culturally sensitive and because the questionnaire was not self-administered.

New item: “*…how much did you worry that your medications will not work due to not eating adequate food?*”

During the interviews it became clear that this was a very sensitive item. All of the twenty participants responded “*not at all*”. They had previously been instructed by the HIV clinic that they must eat well when they started taking antiretroviral medication and so the item appeared to be subject to high levels of social desirability bias. Therefore, the item was removed.

### Measurement equivalence

#### Internal consistency

The internal consistency (Cronbach’s alpha) of the WHOQOL-HIV-BREF-PILOT2 was 0.92. The internal consistencies of the domains were: 0.80, 0.84, 0.70, 0.53, 0.64, and 0.76 for the physical, independence, psychological, spirituality, social, and environment domains respectively. There was no specific item accountable for the low alpha value of the spirituality domain. Two items (“worry about HIV status being found out” and “being accepted by others”) had low corrected item-total correlations both for the whole scale and social domain, and were accountable for the low internal consistency within this domain. When these items were removed the Cronbach’s alpha improved to 0.75. The corrected item total correlation for item on “feeling safe in day to day life” was negative within the whole scale and less than 0.1 with the environment domain. When, this item was removed the internal consistency of the domain became 0.77. The final scale, WHOQOL-HIV-BREF-Eth, had an excellent internal consistency—Cronbach’s alpha of 0.93.

#### Known-groups validity

##### Items

The scores of all the items on WHOQOL-HIV-BREF-PILOT2 were tested for the differences in their scores between the asymptomatic and symptomatic people with HIV. All items except three: “feeling safe in day to day life”, “worry about HIV status being found out”, and “worry about food” demonstrated significant differences in scores between the two groups (Table 3). Therefore, these items were removed so that the final scale, WHOQOL-HIV-BREF-Eth, only has twenty-seven items including the two general items of the original WHOQOL-HIV bref.

**Table 3:** Comparison of median scores of items on WHOQOL-HIV-BREF-PILOT2 between symptomatic and asymptomatic people with HIV in Jimma (n = 100)

Scale/domain and facets	Asymptomatic HIV	Symptomatic HIV	Z score	P value
	Median (IQR)	Median (IQR)		
General QoL	*3 (1)*	*2 (1)*	*3.31*	<*0.001*
General health satisfaction	*4 (1)*	*3 (2)*	*5.80*	<*0.001*
Physical domain				
Pain and discomfort	5 (1)	3 (2)	5.51	<0.001
Symptoms of HIV	5 (2)	3 (3)	5.11	<0.001
Energy and fatigue	4 (2)	3 (1)	5.48	<0.001
Sleep and rest	4 (2)	3 (2)	4.59	<0.001
Independence domain				
Dependence on medication	4.5 (2)	3 (2)	4.03	<0.001
Mobility	5 (1)	3 (2)	5.69	<0.001
Activities of living	4 (1)	2.5 (1)	6.15	<0.001
Work capacity	4 (2)	2 (1)	5.931	<0.001
Psychological domain				
Positive feelings	4 (2)	3 (2)	4.26	<0.001
Self-esteem	5 (1)	3 (2)	5.28	<0.001
Negative feelings	5 (1)	4 (1)	3.30	<0.001
Spirituality domain				
Spiritual	5 (1)	3 (1)	5.58	<0.001
Forgiveness	5 (2)	4 (4)	2.54	<0.05
Fear of the future	5 (2)	3 (3)	3.02	<0.01
Death and dying	5 (0)	3.5 (3)	3.42	<0.001
Social domain				
*Disclosure of HIV status*	*5 (2)*	*4 (3)*	*0.44*	*0.661*
*Ability to support one’s family*	*3 (3)*	*2 (3)*	*3.19*	<*0.01*
Social inclusion	4 (1)	3 (2)	3.17	<0.01
Personal relationships	4 (2)	3 (2)	3.53	<0.001
Social support	4 (2)	3 (2)	2.96	<0.01
*Community relationships*	*4 (1)*	*3 (1)*	*3.73*	<*0.001*
Environmental domain				
Physical safety and security	3 (4)	3.5 (3)	−0.87	0.384
Physical environment	4 (2)	3 (2)	3.13	<0.01
*Worry about food*	*4 (2)*	*3 (3)*	*0.97*	*0.335*
Opportunities for recreation and leisure	4 (2)	3 (3)	2.60	<0.01
Home environment	4 (2)	3 (2)	2.73	<0.01
Access to health and social care	4.5 (1)	4 (2)	2.96	<0.01
Transport	3 (1)	3 (1)	2.86	<0.01
*Access to adequate food*	*4 (2)*	*3 (2)*	*3.59*	<*0.001*

New items are italicized

##### Domains

The mean QoL scores on WHOQOL-HIV-BREF-Eth in asymptomatic people with HIV were significantly higher than those of symptomatic people with HIV on all the six domains. The differences in the mean scores remained statistically significant (P < 0.001) after controlling for age, sex, and literacy status of the participants (Table 4).

**Table 4:** Domain mean scores and differences in the scores on WHOQOL-HIV-BREF-Eth domains between symptomatic and asymptomatic HIV (n = 100)

Quality of life domain	Total sample	Asymptomatic HIV	Symptomatic HIV	Coefficient (95 % confidence interval)
	Mean (SD^a^)	Mean (SD)	Mean (SD)	
Physical	13.8 (4.3)	16.6 (2.7)	11.0 (3.7)	−5.64 (−7.02; −4.25)
Psychological	14.5 (4.0)	16.6 (2.8)	12.4 (3.8)	−4.10 (−5.54; −2.66)
Social	13.5 (2.3)	14.8 (2.8)	12.1 (2.7)	−3.16 (−4.66; −1.66)
Environment	13.0 (2.4)	13.7 (2.1)	12.4 (2.4)	−2.46 (−3.71; −1.21)
Independence	13.6 (4.4)	16.4 (3.2)	10.7 (3.5)	−5.69 (−7.13; −4.25)
Spirituality	15.0 (3.7)	17.0 (2.8)	13.1 (3.5)	−3.61 (−4.98; −2.24)

^a^Standard deviation

#### Factor analysis

Confirmatory factor analysis was performed to evaluate the overall fit of both WHOQOL-HIV-Eth against the six domain structure of the original WHOQOL-HIV bref. The analysis for a six factor structure of WHOQOL-HIV-BREF-Eth found fit indices of χ^2^ = 659.77 (d.f = 260; p < 0.001), CFI = 0.80, TLI = 0.75 and RMSEA = 0.07. When correlations between residuals of some items were allowed, the model fit indices became χ^2^ = 627.75 (DF = 259; p < 0.001), CFI = 0.82, TLI = 0.77 and RMSEA = 0.06. In the model the loading of each item on its corresponding factor was statistically significant. The standardized factor loading to the second order factor was 0.70 for the physical, 0.83 for independence, 0.89 for the psychological, 0.64 for spirituality, 0.89 for the environmental and 0.75 for the social domains.

## Discussion

Despite a rigorous translation and development process, the bref version of the WHOQOL-HIV exhibited some limitations for use among people with HIV in Ethiopia. In particular, we found some problems in the semantic, item and measurement equivalences which might limit its use in clinical settings. The adaptation of the WHOQOL-HIV-bref to the Ethiopian version of WHOQOL-HIV-BREF-Eth resulted in a more culturally appropriate and valid measure of changes in health-related QoL outcomes among people with HIV in Ethiopia. Furthermore, the WHOQOL-HIV-BREF-Eth addresses aspects of QoL in social and environment domains that were not represented within the original tool. However, the changes made to the original tool may preclude comparison of data from other countries.

The semantic equivalence of the WHOQOL-HIV-bref was acceptable for most of the items. Nonetheless, even after rigorous translation and back-translations as well as an expert consensus meeting, problems with the meaning of some of the questions persisted during the test for item equivalence. Most of the problematic items during translation were also problematic items in the item equivalence suggesting the difficulties might lie within the concept that the items were supposed to address. The need for rewording of questions in the WHOQOL-HIV for use in different cultures has been noted by previous researchers [1]. In particular, the general item asking individuals to rate their “quality of life” presented a semantic challenge. Although literal translation was possible, the interviews revealed that the meaning was not straight forward. Therefore, besides rewording the phrase to something like “goodness of living”, it was essential to have additional explanations to ensure that the respondents understood what the question was intended to measure. The problem of questions not being understood as they were intended to has been described in social science research previously [29]. Unsurprisingly, none of the newly added items that were developed locally had problems in semantic or item equivalence. This is probably because the questions were formulated based on the results from a previous qualitative study into the concept of QoL conducted within the same setting [26].

The use of cognitive interviewing offered an opportunity to revise the wording of the questions so that they were understood better [29]. Although it was not possible to assess operational equivalence in this study, the face-to-face interviews helped to examine the cultural appropriateness of some of the questions. In particular, the question asking about “satisfaction with one’s sex life” made some of the respondents reluctant to continue the remaining part of the interviews despite having good semantic equivalence. Similarly, the item addressing individual’s financial resources had no semantic difficulties. However, the responses are uninformative when everyone responds negatively. Researchers often have difficulty measuring financial income through interviews where respondents tend to under-estimate them. In addition, such items become problematic for subsistence farmers.

In agreement with the results from a multinational pilot study, the items on the physical and independence domains better discriminated between symptomatic and asymptomatic people with HIV than items within the environment domain [9]. The failure of the item on “safety and security” to discriminate between the two groups of people with HIV might be related to problems understanding the question itself. The importance of the newly added item on “worry about HIV status being found out” was also reported by international research [7, 9]. However, the item failed to discriminate between symptomatic and asymptomatic people with HIV suggesting that the extent of concern about disclosure remained similar at different stages of the disease. Therefore, this item may not be relevant to measure changes in level of QoL in clinical settings or in intervention studies [1].

Food and nutrition was previously reported as an important theme in measuring QoL in developing countries [28] as well as in the Jimma setting [26]. This was particularly relevant as food insecurity is a common basic need issue among people with HIV in Ethiopia [31]. Among the new three items on food and nutrition, only one of them i.e. “satisfaction with access to adequate food and nutrition” had acceptable psychometric properties. The item on “being worried that medication will not work due to not eating adequate food” might still be relevant but lacked operational equivalence in the context of high levels of illiteracy. It is possible that this item could be retained in situations where the questionnaires are self-administered.

The use of measures of internal consistency in the final adaptation process of WHOQOL-HIV-BREF-Eth has improved the reliability of all but the spirituality domain. The latter had an unsatisfactory Cronbach’s alpha perhaps because of the fact that little or no change was made to the items from the original WHOQOL-HIV bref [10]. Findings from the international validation study of the tool also found that the spirituality domain has sub-optimal internal consistency [9]. In the light of the ability of the items in this domain to discriminate between different stages of disease and the corrected item-total correlations being acceptable, it was reasonable to keep the items as they were. Nonetheless, the potential limitation of the spirituality domain within the WHOQOL-HIV-BREF-Eth needs to be recognized.

Previous studies have suggested fewer than six domains for the WHOQOL-HIV bref [13, 22]. The sub-optimal fit indices found on confirmatory factor analysis for a six domain structure may also support that. However, the indices could be acceptable given the item modifications made to the original tool.

In the process of adapting the WHOQOL-HIV bref to the Ethiopian setting, three of the six newly introduced items were subsequently removed. In addition, seven items from the original WHO tool had to be removed due to problems with cultural relevance and acceptability, problems with meaning, or poor psychometric properties. Although such a degree of modification of a tool developed for international use limits its utility for comparisons of results across countries, it is likely to enhance the overall validity of the adapted tool within the given population. The need for such a degree of modification of the WHOQOL-HIV bref might have resulted from underrepresentation of sub-Saharan African populations during the development and piloting of the WHOQOL-HIV [7, 8].

We believe that the adapted version, WHOQOL-HIV-BREF-Eth, has acceptable validity for use among Ethiopian people with HIV in clinical settings. First, the adapted tool has demonstrated excellent known-groups validity across all the six domains making it ideal to measure intervention outcomes among people with HIV. Second, the internal consistency of five of the six domains ranged from acceptable to very good despite the introduction of the new items. Third, the addition of new items generated from a previous qualitative study in the same population addresses aspects of QoL that are important for people with HIV in this Ethiopian setting.

This study has attempted to address most of the elements of a standardized approach to cross-cultural validation of a QoL assessment tool suggested by experts [1, 12]. Our approach has implemented a rigorous translation process to ensure semantic equivalence, and a detailed assessment of item equivalence. Also, the study has attempted to address the lack of representation of relevant aspects of QoL which were identified by previous researchers [26, 28]. Nonetheless, the study is not without limitations. First, the relatively small sample size limited the assessment of construct validity through exploratory factor analysis. The latter has hindered the identification of the optimal number of factors for analysis in the confirmatory factor analysis. Second, by conducting the study at one site, the generalizability might be limited in a country like Ethiopia which has a diverse population. Third, the cross-sectional design precludes the assessment of the tool’s responsiveness to change. Finally, the face-to-face interviewing method might have led to the poor psychometric properties of some of the items which might function well if the participants had filled out the questionnaires on their own. However, in settings with low literacy this approach is not possible.

## Conclusions

Many aspects of QoL appear to be relevant cross-culturally. The rigorous process of adaptation of the WHOQOL-HIV-BREF-Eth indicates its validity for use among people with HIV in Ethiopia.

## Abbreviations

CFI: comparative fit index
DF: degrees of freedom
HIV: human immunodeficiency virus
QoL: quality of life
RMSEA: root mean square error approximation
SD: standard deviation
TLI: Tucker-Lewis index
WHO: World health Organization
WHOQOL: World Health Organization quality of life
WHOQOL-HIV: World Health Organization quality of life—HIV version
